# The Association between Noise Exposure and Metabolic Syndrome: A Longitudinal Cohort Study in Taiwan

**DOI:** 10.3390/ijerph17124236

**Published:** 2020-06-14

**Authors:** Tao Huang, Ta-Chien Chan, Ying-Jhen Huang, Wen-Chi Pan

**Affiliations:** 1 Research Center for Humanities and Social Sciences, Academia Sinica, Taipei 100029, Taiwan; tao7753@gmail.com (T.H.); juuy231282@gmail.com (Y.-J.H.); 2 Institute of Public Health, School of Medicine, National Yang-Ming University, Taipei 11221, Taiwan; 3 Institute of Environmental and Occupational Health Sciences, National Yang-Ming University, Taipei 11221, Taiwan; wenchipan@post.harvard.edu

**Keywords:** noise exposure, human perception, metabolic syndrome, hazard ratio

## Abstract

Metabolic syndrome is becoming more common worldwide. Studies suggest environmental pollution, including traffic noise, might be linked with metabolic syndrome. This study sought to evaluate how noise exposure is linked to the development of metabolic syndrome and its components in Taiwan. Using data from a cohort of 42,509 participants and Cox proportional hazards regression models, the effects of noise exposure on metabolic syndrome and its components were quantified. After adjustment for covariates (age, gender, body mass index, and physical activity), the hazard ratio for metabolic syndrome was 1.13 (95% CI: 1.04–1.22) for medium noise exposure and 1.24 (95% CI: 1.13–1.36) for high noise exposure. Noise exposure was also positively associated with all of metabolic syndrome’s components. This finding suggests noise exposure might contribute to metabolic syndrome and its components. Policies aiming to reduce noise pollution might reduce the risks of metabolic syndrome and its components.

## 1. Introduction

Metabolic syndrome is a cluster of physiological risk factors, and has five components, including abdominal obesity, hypertension, hyperglycemia, hypertriglyceridemia, and low high-density lipoprotein (HDL) cholesterol. Metabolic syndrome is associated with the incidence of cardiovascular diseases [1] and cancers [2]. It is becoming more common around the world [3,4], and, in most Asia-Pacific countries, nearly one-fifth of the adult population are affected by metabolic syndrome [5].

Noise annoyance is associated with road, rail, aircraft, ship, and industrial noise exposure. Sources of noise pollution have different characteristics and impacts on human health. Road noise is associated with negative human health effects, including a wider waist circumference and a higher BMI [6]. Morley et al. [7] and Ruiz-Padillo et al. [8] suggested using traffic flow and traffic composition to model road noise for noise control actions. Thiesse et al. [9] found an association between railway noise exposure and a higher risk of impaired glucose tolerance and insulin sensitivity. Moreover, people are disturbed by vibration-induced railway noise [10] and by train horns [11]. Aircraft overflights may impact rural and distant quiet areas, causing noticeable soundscape degradation [12]. Stansfeld et al. [13] identified associations between aircraft noise exposure and health effects in terms of reading comprehension, recognition memory and annoyance among 3207 children. Aircraft noise is more annoying than road and rail traffic at the same Lden level [14]. Wind turbine noise has indirect health effects including sleep disturbance annoyance [15]. The low-frequency noise generated by wind turbines can induce vibration in building structures [16], and the noise annoyance the turbines cause is higher than road, rail, and aircraft noise [17,18]. Finally, ship and port noise are less regulated compared to other noise sources [19,20], and vessels moving in canals in a city center or ports near metropolitan areas may generate noise pollution and have harmful impacts on urban populations [21,22].

With increasing urbanization, exposure to environmental stressors such as noise and air pollution is increasing. Noise exposure is heterogeneously distributed, and social inequalities in noise exposure have been identified. One study found that areas with higher black and Hispanic populations are associated with increased road and air traffic noise compared to those with more predominantly white, non-Hispanic populations [23]. Noise exposure is usually higher in metropolitan areas than in rural areas. Road noise exposure has been found to decrease along an urban–rural gradient (metropolitan areas, micropolitan areas, small towns, and rural areas) in the United States [24]. Another study has also found that metropolitan areas had higher noise exposure, regardless of racial composition [25]. Based on a previous report, over 90% of the Tainan City (Taiwan) population are exposed to unacceptable noise [26]. Environmental noise represents a potential metabolic syndrome risk factor. In comparison with studies on the impact of noise on the cardiovascular system, the number of studies on its impact on the metabolic system has been limited [27]. One study found a link between noise exposure and elevated risk of metabolic syndrome in elderly Mexican Americans [28]. However, no studies have focused on Asians, despite the increasing prevalence of metabolic syndrome in the Asia-Pacific region.

Noise can cause not only auditory health effects, but also non-auditory ones. Although the strength of the associations varies significantly across studies, chronic exposure to noise is associated with elevated blood pressure and other metabolic outcomes. In response to noise, stress hormones are released [29]. Psychophysiological stress reaction to noise is associated with metabolic and cardiovascular outcomes [30,31]. The suggested mechanisms include an effect of noise-induced stress on alterations in glucose and insulin regulation [9].

Previous studies have found associations between noise exposure and metabolic syndrome and its components, but the associations are inconsistent among cohort studies. One study suggested that traffic-related NOx and noise elevated the risk of having metabolic syndrome and low HDL cholesterol in older Mexican Americans in the Sacramento area [28]. Another study found an association between long-term noise exposure and general obesity in women in Norway [32]. Arlien-Søborg et al. [33] found that occupational noise exposure levels are strongly associated with increasing levels of triglycerides and cholesterol HDL ratio, and a decreasing level of HDL cholesterol. In addition, higher exposure to black carbon (a marker of traffic-related pollution) has been shown to be significantly associated with low HDL cholesterol [34]. Aircraft noise exposure at nighttime may increase the risk of hypertension in men [35].

A noise map is an efficient tool to estimate an individual’s noise exposure. Noise maps based on sound pressure levels may not represent how people perceive the sound environment. Noise perception is associated with environmental noise exposure [36,37]. Researchers have suggested using soundscape maps based on human perception of the sound environment to represent noise exposure if a large amount of perceptual data is available [38]. Previous studies have assessed the effects of perceived noise exposure on residents’ health. A Swiss cohort study indicated that perceived transportation noise, but not measured transportation noise (Lden), was associated with respiratory symptoms and current asthma in adults [39]. Traffic noise perception contributed to stress and was negatively associated with self-reported mental health status in an Australia cross-sectional study [40].

Spatial analysis of environmental perception data can be used to explore coupled relationships of human and environmental systems [41]. According to Tobler’s first law of geography, “everything is related to everything else, but near things are more related than distant things” [42]. The geographic distribution of noise monitoring stations is sparser than the perception points in our study area. Perception is better than measurements to estimate noise maps, due to a higher density of measured points. Geostatistical interpolation is an approach to estimate pollution levels at unsampled locations. Several studies have used spatial interpolation to predict exposure to pollution at the individual level [43]. Kriging, one of the most widely used interpolation methods, was applied to estimate noise exposure at the individual level in this study. The advantage of Kriging is that it predicts values at unsampled locations and estimates the standard errors of predictions. The error estimates inform us of where predicted values are less reliable [44]. For a better representation of noise exposure, this study used ordinary Kriging to generate the perceived noise map from 50,720 health screening records in 2014 and 2015.

Long-term effects of noise on the development of metabolic syndrome have not been studied in Asia. The aim of this study was to quantify the association between noise exposure and the development of metabolic syndrome. This study hypothesized that noise exposure increases the risk of metabolic syndrome. To test this hypothesis, Cox regression models were applied to quantify the effects of noise exposure on metabolic syndrome and its components in a cohort in Taiwan.

## 2. Materials and Methods

**Figure 1:**
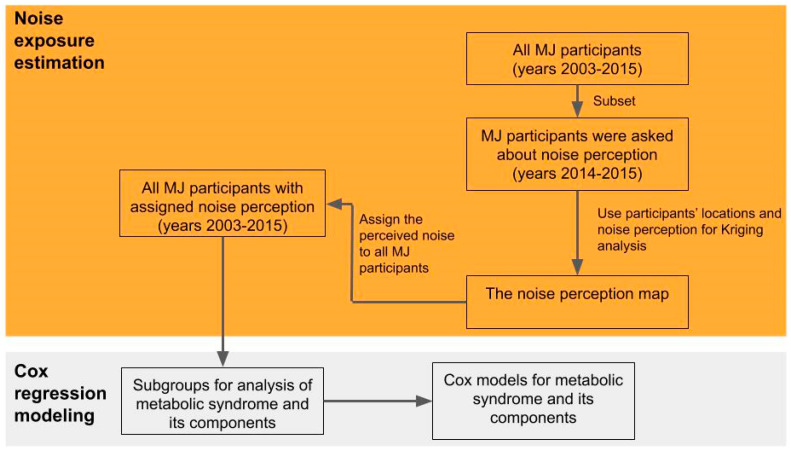
Flowchart of this study. This study first estimated the noise exposure for each MJ health screening participant, then Cox regression models were applied to estimate the hazard ratios of metabolic syndrome and its components under different levels of noise exposure.

The overarching approach of this study includes two parts: noise exposure estimation for each participant and determining the association between noise exposure and metabolic syndrome and its components (Figure 1). The details of the study participant description, noise mapping, and Cox regression model parameterization are described below.

### 2.1. Ethics

This study was approved by the Institutional Review Board (IRB) of Biomedical Science Research, Academia Sinica (AS-IRB-BM 17044). This study was designed as a retrospective longitudinal cohort study. Each participant gave written consent prior to participation to authorize the use of data generated from the medical examination program. Personal identification was removed, and the data remained anonymous when released for research purposes.

### 2.2. Study Participants

**Figure 2:**
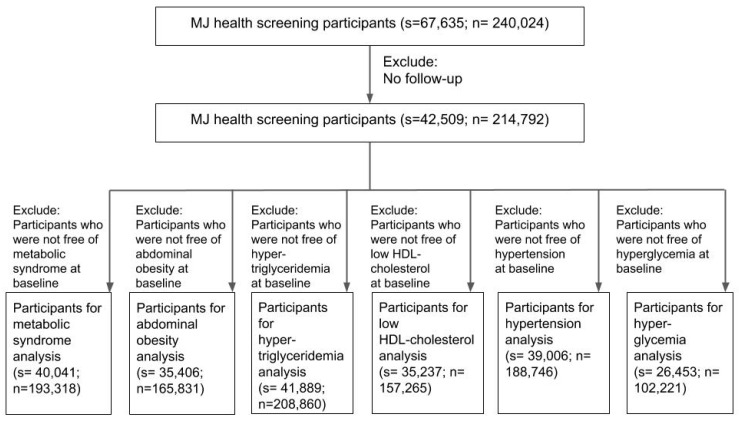
Participant flow diagram illustrating the exclusion criteria for analysis of metabolic syndrome and its components (s: participants, n: records).

The cohort was established by the Mei Jau (MJ) Health Management Institution, which is a private firm offering a comprehensive health screening program. A total of 67,635 MJ health screening participants were followed from 2003 to 2015. The following were the exclusion criteria for the participants: (1) no follow-up data available and (2) being diagnosed as having metabolic syndrome or its components at the baseline. The process for exclusion of participants is shown in Figure 2. A total of 42,509 participants were included for further analysis after excluding participants lacking follow-up visits. The participants were classified as having metabolic syndrome if they had three or more of the following five components: abdominal obesity, hypertension, low high-density lipoprotein, hypertriglyceridemia, and hyperglycemia. 

According to the criteria of the Ministry of Health and Welfare, Taiwan, the criteria of metabolic syndrome for Taiwanese require three or more of the following measurements: (1) abdominal obesity: waist circumference >90 cm (35 inches) for men and >80 cm (31 inches) for women [45]; (2) hypertension: raised blood pressure (BP): systolic BP ≥ 130 mmHg or diastolic BP ≥ 85 mmHg or current use of antihypertensive drugs. Instead of applying the blood pressure data from the MJ database, this study used the long-term use of antihypertensive drugs as the proxy variable; (3) hyperglycemia: raised fasting plasma glucose (FPG): FPG ≥ 100 mg/dL or current use of antihyperglycemic drugs [46]; (4) hypertriglyceridemia: higher triglycerides (TG): TG ≥ 150 mg/dL or current use of antitriglyceride drugs; (5) lower high-density lipoprotein cholesterol (HDL-C): HDL-C <40 mg/dL for men and <50 mg/dL for women [47]. Participants were excluded if they had metabolic syndrome or its component at the baseline or if no follow-up data was available (Figure 2). Most participants lived in metropolitan areas (Figure S1).

### 2.3. Noise Mapping

To create the noise exposure map, this study used the participants’ noise perception as the input to perform spatial interpolation analysis. Data was collected by means of a questionnaire about the participants’ noise perception administered during MJ health screening in 2014 and 2015. Participants were asked to evaluate the degree of noise pollution within a radius of 1.5 km of their homes. Noise perception was the degree of annoyance (on a scale of 1–4, where 1: extremely serious, 2: serious, 3: not serious, and 4: not serious at all). The geostatistical Kriging approach was used to create a noise perception map. Ordinary Kriging is a linear geostatistical interpolation technique which has been used for sound level mapping (e.g., [48]). This study used ArcGIS (ArcMap, version 10.5; ESRI Inc., Redlands, CA, USA) with the extension of Spatial Analyst to perform Kriging analysis. Participants’ locations and the corresponding perceptions were used as the input for Kriging analysis. Root-mean-square error (RMSE) was used to evaluate the accuracy or best fit of the Kriging tool. This study chose the stable model as it had the lowest RMSE. Perceived noise is not directly comparable to measured noise. Categorized noise perception was compared with the measured noise for the model validation. Seasonal noise information was retrieved from 423 noise-monitoring stations maintained by the Taiwan Environmental Protection Administration in 2003–2015. This study excluded 164 stations where no MJ participants lived within 500 m of the noise stations. Noise data from the 259 stations was further aggregated in an annual fashion for the analyses. Mean measured noise for each noise station was calculated. The perceived noise of each noise measurement station was calculated by averaging the noise perceptions of participants within 500 m of the noise stations. 

### 2.4. Statistical Analysis

The Cox proportional hazards model was applied with the visiting date as the underlying time variable to estimate the hazard ratios for metabolic syndrome and its components under different noise exposure levels. The Cox proportional hazards model has been widely used in analysis of time-to-event data [49]. For metabolic syndrome and each of its components, a univariate model (noise perception as the single variable) and a multivariable model (adjusted for baseline age, body mass index (BMI), gender, and physical activity) were developed. The model was used to estimate hazard ratios (HRs) and their 95% CIs for evaluating the associations between noise exposure and metabolic syndrome. The hazard ratio is defined as the ratio of the risk of the event of interest (e.g., death) in one group to the risk of the event of interest in the other group occurring at a given interval of time [50]. The selection of covariates was done based on existing literature. All statistical analyses were performed using R 3.6.3 [51], and the model was fitted with the coxph function in the survival package.

### 2.5. Perceived Noise Exposure and Covariates

Noise perception was estimated at each participant’s geocoded residential address. Perceived noise for each participant was categorized into high (perception < 1st quartile), medium (3rd quartile ≥ perception ≥ 1st quartile), and low (perception < 3rd quartile). The noise perception was only available in 2014 and 2015. Therefore, this study assumed the spatial distribution of perception was consistent from 2003 to 2015 based on a cross-check of real noise measurements. Body mass index (BMI) was stratified into underweight (BMI < 18.5), normal (18.5 ≤ BMI ≤ 24), and overweight (BMI > 24) [52]. Age was stratified into 0–39, 40–59, and ≥60 years. Physical activity was assessed by a questionnaire at every visit. Participants were asked to report their weekly physical activity under four intensity categories: light (e.g., walking), moderate (e.g., playing basketball), medium vigorous (e.g., jogging), and high vigorous (e.g., running). A metabolic equivalent (MET) value (1 MET ≥ 1 kcal/hour/kg) was assigned to each intensity category as follows: 2.5 METs for light, 4.5 METs for moderate, 6.5 METs for medium vigorous, and 8.5 METs for high vigorous. A total of 7.5 MET-h/wk is required for health benefits, and twice that level, 15.0 MET-h/wk, for additional benefits [53]. A total of 7.5 MET-hours/week is required to achieve the minimum level of the WHO-recommended leisure time physical activity [54]. The participants were classified into one of the following categories using the cut-points of 3.75 MET-hours/week (half of the recommended level) and 7.5 MET-hours/week (recommended level) [55]. 

## 3. Results

### 3.1. Noise Mapping

**Figure 3:**
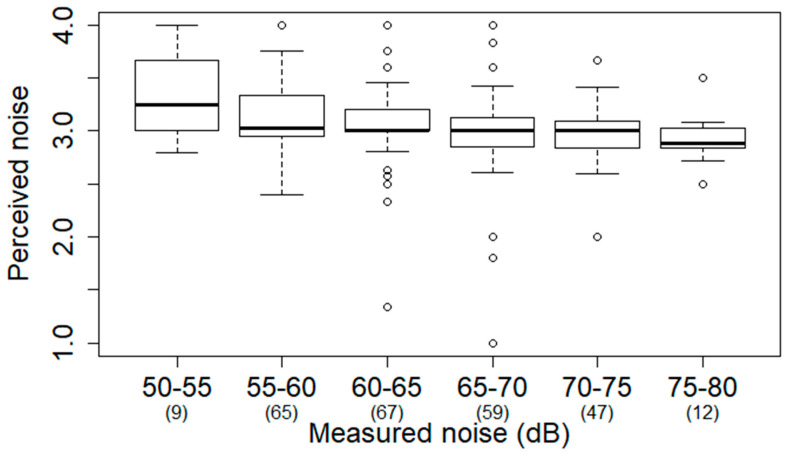
Perceived noise (scale of 1–4 where 1 is extremely serious, 2 is serious, 3 is not serious, and 4 is not serious at all) within 500 m of noise monitoring stations increased with measured noise. The numbers of stations in each measured noise category are shown in parentheses.

The estimated perceived noise map is shown in Figure S2. The Kriging model was evaluated using cross-validated estimators of root-mean-square error (RMSE). The RMSE is 0.57. The perceived noise was slightly higher (mean: 3.17; standard deviation: 0.26; scale of 1–4 where 1 is extremely serious, 2 is serious, 3 is not serious, and 4 is not serious at all) in the major metropolitan areas (Taipei, Taoyuan, Hsinchu, Taichung, Chiayi, Tainan, and Kaohsiung) than the perceived noise (mean: 3.29; standard deviation: 0.25) in rural areas. Kriging-interpolated prediction surface standard error shows that metropolitan areas, where most health screening participants were located (Figure S1), had lower standard error than rural areas (Figure S3). This suggests the perceived noise estimation is reliable for our analysis. Figure 3 shows the mean noise perception of participants within 500 m of the noise stations versus the mean measured noise of noise stations. In general, participants perceived higher noise when measured noise was higher.

### 3.2. Baseline Characteristics of the Study Participants

Baseline characteristics of the study population are shown in Table 1. At baseline, the gender ratio is close to 1:1 except for the cohort of hyperglycemia (male: 42.1%; female: 57.9%). The average age of the participants in each sub-group was approximately 40 years. The average body mass index (BMI) was 21–23. The metabolic equivalent (MET) was approximately 8 hrs/wk. The estimated noise perception was approximately 3 (not serious).

### 3.3. Association of Perceived Noise Exposure and Metabolic Syndrome

Noise exposure was linked to a statistically significantly higher risk of metabolic syndrome. The hazard ratio of metabolic syndrome in comparison with the reference category was 1.13 (95% CI: 1.04–1.22) for medium and 1.24 (95% CI: 1.13–1.36) for high noise exposure (Figure S4). Although noise was not significantly associated with metabolic syndrome in the univariate model, noise exposure was significantly associated with metabolic syndrome in the models that adjusted for gender, age, BMI, and physical activity (MET) (Table 2). Among all covariates, overweight BMI had the highest hazard ratio (Figure S4). Age was also positively associated with metabolic syndrome. Our analyses suggested that females had higher risks of metabolic syndrome. MET was the only variable with a hazard ratio smaller than 1, suggesting physical activity protects against metabolic syndrome.

### 3.4. Association of Perceived Noise Exposure and Metabolic Syndrome Components

**Figure 4:**
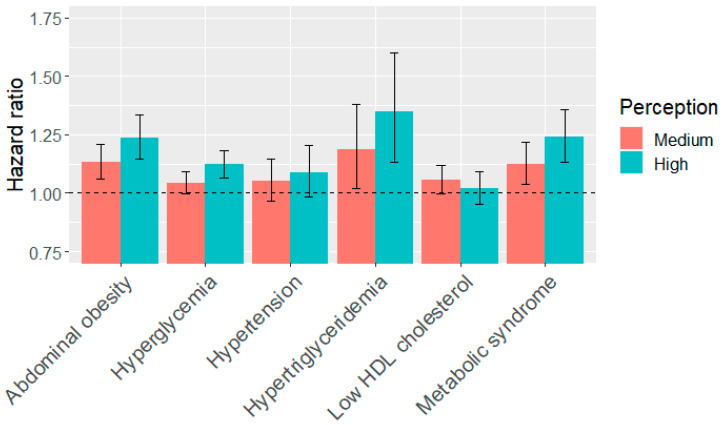
Effect estimates (and 95% confidence intervals) from multivariate Cox models of metabolic syndrome and its components.

In general, noise exposure was positively associated with all components of metabolic syndrome. For the univariate model, elevated noise exposure was positively associated with the new occurrence of four components of metabolic syndrome, namely, abdominal obesity, hyperglycemia, hypertriglyceridemia and low HDL cholesterol during follow-up, but negatively with hypertension (Table 2). For the multivariate models, noise exposure was positively associated with all components of metabolic syndrome (Table 2). This study found that the risks of hypertension and low HDL cholesterol were associated with noise exposure. However, these associations did not attain statistical significance (Figure 4). The hazard ratio of hyperglycemia significantly increased only at the high noise exposure level (Figure 4). BMI was positively associated with all of metabolic syndrome components (Figures S5–S9). The hazard ratio for each metabolic syndrome component was the highest in the old age category, except for low HDL cholesterol (Figures S5–S9). The hazard ratios of abdominal obesity and low HDL cholesterol were higher for females than males, while hazard ratios of hyperglycemia, hypertension, and hypertriglyceridemia were higher for males than females (Figures S5–S9). For physical activity, the hazard ratios for all metabolic syndrome components were smaller than 1 in the high-MET category (Figures S5–S9).

## 4. Discussion

This is one of few studies examining the relationship between noise exposure and metabolic syndrome and its components. This study found noise exposure was associated with a significantly increased risk of metabolic syndrome after controlling for a wide range of covariates. The associations between noise exposure and each metabolic syndrome component were also consistently stronger among persons with medium or high noise exposure than persons with low noise exposure. Noise exposure was associated with significantly increased risks of hypertriglyceridemia, abdominal obesity, and hyperglycemia, while the hazard ratios for noise on hypertension and low HDL cholesterol were lower and not statistically significant.

Effects of long-term noise exposure on the metabolic system have only recently been addressed in epidemiological research. Our findings are consistent with the results from a previous study (Yu et al. [28]) focusing on elderly Mexican American participants. They divided the noise exposure level into four categories and found the risk of metabolic syndrome was significantly higher in the highest noise category. However, their study was limited to a smaller number of elderly participants (*n* ≥ 1554). A longitudinal study of an ageing population of the Whitehall II study, UK (aged 45–69 years at baseline) [56] observed that greater long-term exposure to greenspace surrounding one’s residence was associated with a lower risk of developing metabolic syndrome. The authors suggested that noise may be an important mechanism underlying the association between long-term greenspace exposure and metabolic syndrome. Although the mechanisms behind chronic effects of noise on the metabolic system are not fully understood, there are several plausible pathways [57]. For example, noise-induced sleep deprivation may have a long-term metabolic consequence.

This study also found significant associations between noise and hypertriglyceridemia. The association of residential noise and hypertriglyceridemia has been reported in previous studies. One study found noise exposure was positively associated with hypertriglyceridemia, but the 95% CIs crossed the null [28]. In a study of 144,082 participants aged ≥ 20 years, Cai et al. [58] found higher daytime noise exposure was positively associated with higher triglycerides. Besides, one study found an association of occupational noise and increasing triglyceride levels [33]. Another study found the triglycerides of a group exposed to high industrial noise were significantly higher than a low-exposure group after adjustment for age, BMI, smoking and work hours per week [59].

Our findings indicate that noise exposure was associated with a significantly increased risk of abdominal obesity. Our results are corroborated by some previous studies. A Swedish study observed an association between road traffic noise and abdominal obesity, which was statistically significant only in women [60]. Another study also found that long-term exposure to road traffic noise over time may increase the risk of obesity [61]. In contrast, one study did not find positive associations between traffic-related noise exposure and abdominal obesity [28]. In that study, the incidence rate for abdominal obesity (30.4%) was lower than other metabolic syndrome components (34.4−68.1%). The authors suggested that this was because the average participants in their study were 70 years old at enrollment, and that was too late for abdominal obesity to be newly occurring.

This study found the risk of hyperglycemia was significantly increased at the high noise exposure level. This is consistent with previous studies. One study of a population of 3350 adults aged 29–81 years in Switzerland [62] found positive associations between exposure to night-time road traffic noise and three-month average glycemia. Another study found that subjects exposed to occupational noise levels >80 dBA had a significantly higher risk of hyperglycemia compared with those exposed to <70 dBA [63]. One birth cohort study [64] found that exposure to green space was associated with lower maternal blood glucose, and suggested that reduced levels of noise exposure might be a potential mechanism linking green space to maternal blood glucose outcomes.

No significant association between noise and low HDL cholesterol was found in our study. Another research group also found no significant association between road traffic noise and low HDL cholesterol in a population of students in Slovakia [65]. Although Yu et al. [28] suggested that low HDL cholesterol risk was increased by NOx exposure, not by noise exposure, another study found that it was difficult to separate effects of air pollution and noise in relation to blood cholesterol [66].

The present study observed that the hazard ratio of hypertension was associated with noise exposure, but these associations did not attain statistical significance. Some previous studies found noise exposure increased blood pressure, but not the risk of hypertension. Although one study found a positive association of systolic blood pressure and road traffic noise levels [67], it did not find associations with hypertension. The use of a categorical variable (for hypertension) might lead to a loss of information, or the risk of hypertension might increase only above a certain noise threshold. Another study, on occupational health, observed that, as workers were exposed to noise over 80 dBA, the hazard ratios of hypertension significantly increased [68]. Noise exposure has been found to be associated only with clinically more severe hypertension, but not with uncomplicated hypertension [69]. The current understanding of the underlying mechanisms suggests that noise activates the hypothalamic–pituitary–adrenal axis (HSA) and the sympathetic–adrenal–medullar axis (SAM) to induce an increase in stress hormones (e.g., catecholamines), which play a major role in blood pressure regulation [70,71].

This study has some important strengths, including its use of a large dataset. However, there are also several limitations. One is possible misclassification of traffic noise exposure since noise exposure was estimated from noise perception data. However, this concern is reduced by the fact that the perceived noise shows trends similar to the measured noise. Another limitation is that the perceived noise exposure levels were calculated at the fixed locations of the participants, and their daily activity patterns were not considered. Noise perception data was not available before 2014. Thus, this study assumed that perception and exposure were constant with time. Although the spatio-temporal trend of the noise measurement has been examined, the local variations with time might still be underestimated. Moreover, the follow-up intervals in this study varied from participant to participant. Participants’ dietary composition was not included in our model because (1) the food frequency questionnaire had only 15 questions, which is lower than the typical number of items, 80–120 [72]; (2) the dietary questionnaire characterized the diet information in the past one month and may be subject to recall bias; and (3) 5.6% of records had incomplete dietary records. A more comprehensive dietary questionnaire, including cooking methods, types of meats, and more food groups, is needed to include dietary composition in the analysis. Other environmental factors, including air pollution, might also increase the risk of metabolic syndrome. Future studies should take air pollution into consideration.

## 5. Conclusions

Prevention of metabolic syndrome is one of the main public health priorities of the 21st century. This study first created a map of perceived noise exposure, then derived noise perception from the noise map to determine the association between noise exposure and metabolic syndrome and its components. The results indicate that noise significantly increased the risk of metabolic syndrome and was positively associated with the risk of all components of metabolic syndrome after adjusting for age, BMI, gender, and physical activity in Taiwan. To improve the understanding of associations between noise exposure and metabolic syndrome and its components, future studies are needed to include other metabolic syndrome risk factors, including air pollution, and investigate the underlying mechanisms. This study stresses the need to regulate environmental noise so as to mitigate negative health consequences. An implication for decision makers is that reducing traffic noise may also reduce metabolic syndrome and generate large public health benefits. Residents’ noise perceptions should be taken into account when designing healthy communities.

## Figures and Tables

**Table 1:** Baseline characteristics of the MJ participants analyzed for metabolic syndrome and its components.

	Metabolic Syndrome Mean (SD) or % (*n*)	Low HDL Cholesterol Mean (SD) or % (*n*)	Abdominal Obesity Mean (SD) or % (*n*)	Hyperglycemia Mean (SD) or % (*n*)	Hypertriglyceridemia Mean (SD) or % (*n*)	Hypertension Mean (SD) or % (*n*)
Gender	M: 49.6% (19,860) F: 50.4% (20,181)	M: 49.9% (17,571) F: 50.1% (17,666)	M: 48.0% (17,002) F: 52.0% (18,404)	M: 42.1% (11,068) F: 57.9% (15,219)	M: 49.7% (20,823) F: 50.3% (21,066)	M: 49.4% (19,277) F: 50.6% (19,729)
Age, years	41 (13)	41 (13)	40 (12)	38 (12)	41 (13)	40 (12)
Body mass index, kg/m^2^	22.6 (3.2)	22.5 (3.3)	21.9 (2.7)	22.1 (3.3)	22.8 (3.5)	22.6 (3.3)
Physical activity, MET hrs/wk	8.3 (14.2)	8.5 (14.3)	8.3 (14.2)	7.7 (13.1)	8.4 (14.2)	8.2 (14.1)
Perceived noise	3.0 (0.19)	3.0 (0.20)	3.0 (0.19)	3.0 (0.19)	3.0 (0.20)	3.0 (0.19)

**Table 2:** Effect estimates (and 95% CIs) from Cox models.

		Univariate Model ^1^			Multivariable Model ^2^		
Risk Factor (events/subjects)	Noise Exposure	HR	CI	*p*-Value	HR	CI	*p*-Value
Metabolic syndrome (3804/40,041)	Medium perception	1.0	0.93–1.1	0.863	1.13	1.04–1.22	0.003
	High perception	1.1	0.98–1.2	0.129	1.24	1.13–1.36	<0.001
Low HDL cholesterol (7197/35,237)	Medium perception	1.1	1.01–1.1	0.015	1.06	1.00–1.12	0.054
	High perception	1.0	0.97–1.1	0.231	1.02	0.95–1.09	0.576
Abdominal obesity (5778/35,406)	Medium perception	1.1	1–1.1	0.069	1.13	1.06–1.21	<0.001
	High perception	1.1	1–1.2	0.002	1.24	1.15–1.33	<0.001
Hypertension (3167/39,006)	Medium perception	0.93	0.86–1	0.1	1.05	0.97–1.14	0.235
	High perception	0.94	0.86–1	0.245	1.09	0.99–1.20	0.088
Hyper-triglyceridemia (1090/41,889)	Medium perception	1.1	0.94–1.26	0.281	1.19	1.02–1.38	0.023
	High perception	1.2	1.01–1.42	0.034	1.35	1.14–1.60	0.001
Hyperglycemia (11,273/26,453)	Medium perception	1.0	0.95–1.0	0.937	1.04	1.00–1.09	0.059
	High perception	1.1	1.00–1.1	0.055	1.12	1.07–1.18	<0.001

CI, confidence interval; HR, hazard ratio. ^1^ Noise perception is the only variable. ^2^ Adjusted for baseline age, gender, baseline BMI, and physical activity.

## Supplementary Materials

The following are available online at https://www.mdpi.com/1660-4601/17/12/4236/s1, Figure S1. Distribution of the MJ participants for the years 2003–2015. Figure S2. Perceived noise map (scale of 1–4 where 1 is extremely serious, 2 is serious, 3 is not serious, and 4 is not serious at all). Figure S3. Standard error map of predicted perceived noise. Figure S4. Hazard ratio (and 95% confidence intervals (CI)) of metabolic syndrome (R2 ≥ 0.71). Figure S5. Hazard ratio (and 95% confidence intervals (CI)) of hypertriglyceridemia (R2 ≥ 0.55). Figure S6. Hazard ratio (and 95% confidence intervals (CI)) of low HDL cholesterol (R2 ≥ 0.16). Figure S7. Hazard ratio (and 95% confidence intervals (CI)) of hypertension (R2 ≥ 0.50). Figure S8. Hazard ratio (and 95% confidence intervals (CI)) of hyperglycemia (R2 ≥ 0.23). Figure S9. Hazard ratio (and 95% confidence intervals (CI)) of abdominal obesity (R2 ≥ 0.68).
