# Supplementary material for: The Association between Noise Exposure and Metabolic Syndrome: A Longitudinal Cohort Study in Taiwan
Source: Int J Environ Res Public Health. 2020 Jun 14;17(12):4236. doi: 10.3390/ijerph17124236 (PMC7344493; doi:10.3390/ijerph17124236)
Supplement: Supplementary file 1 [file ijerph-17-04236-s001.pdf]

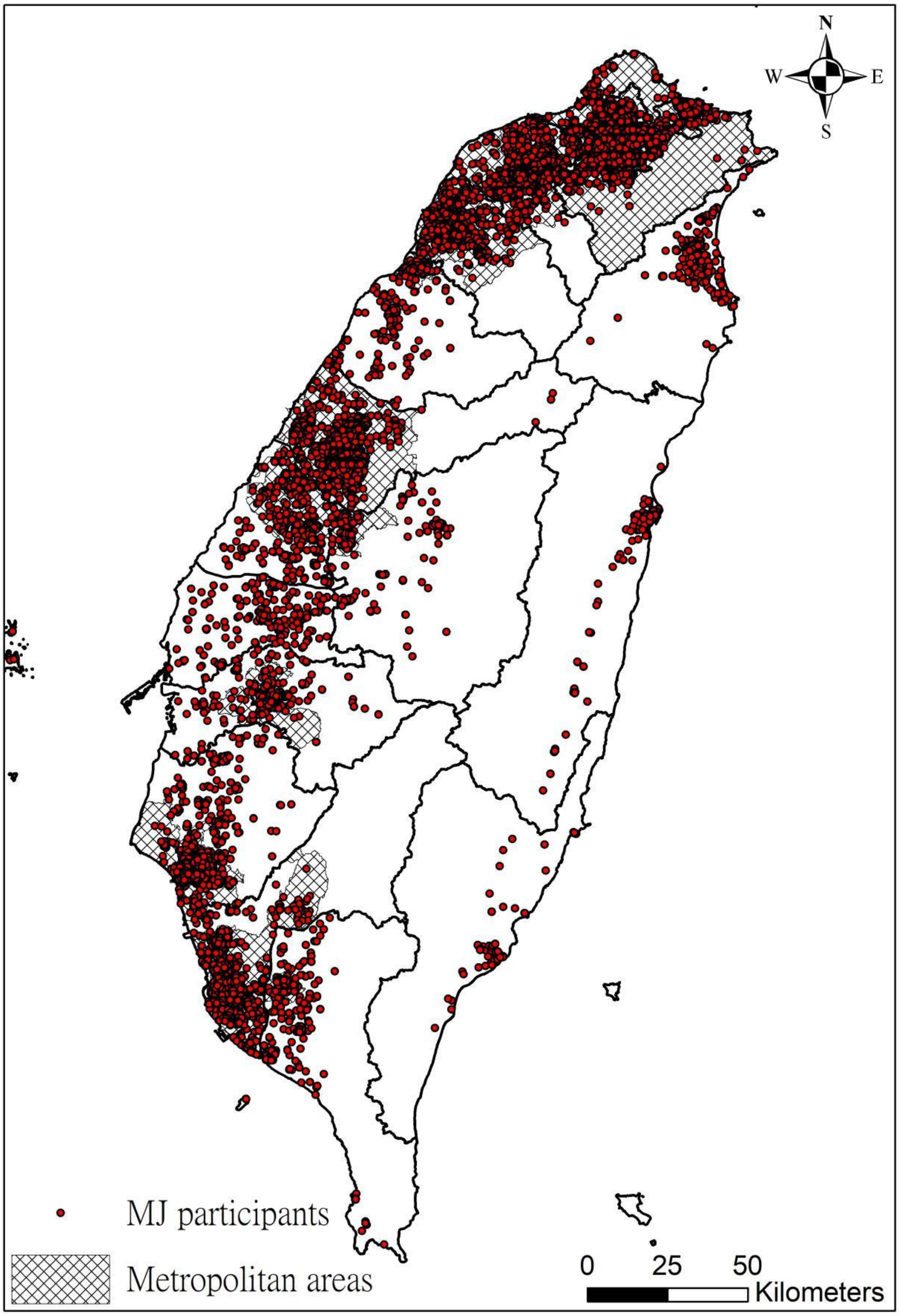

Figure S1. Distribution of the MJ participants for the years 2003-2015.

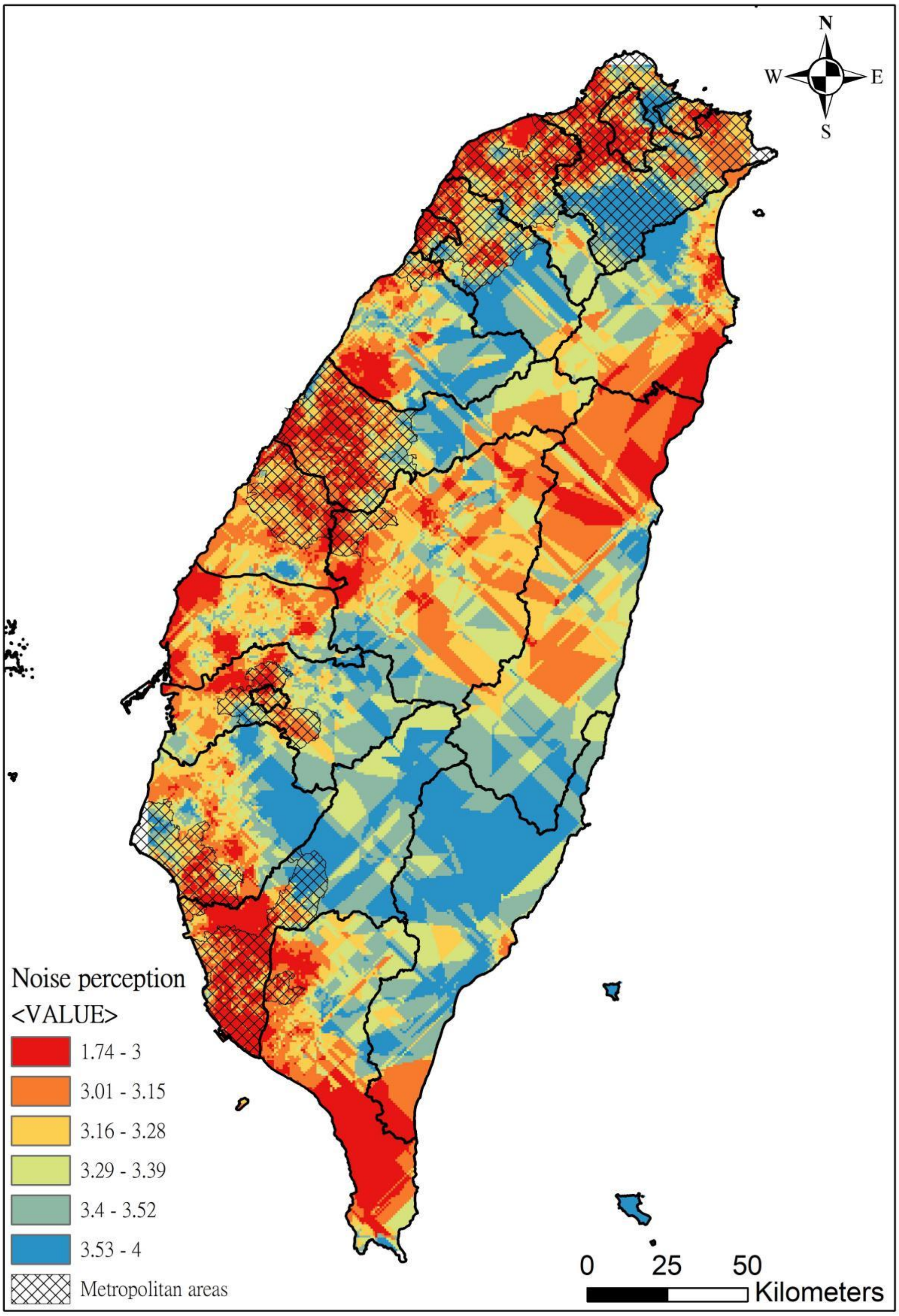

Figure S2. Perceived noise map (scale of 1-4 where 1: extremely serious, 2: serious, 3: not serious, and 4: not serious at all).

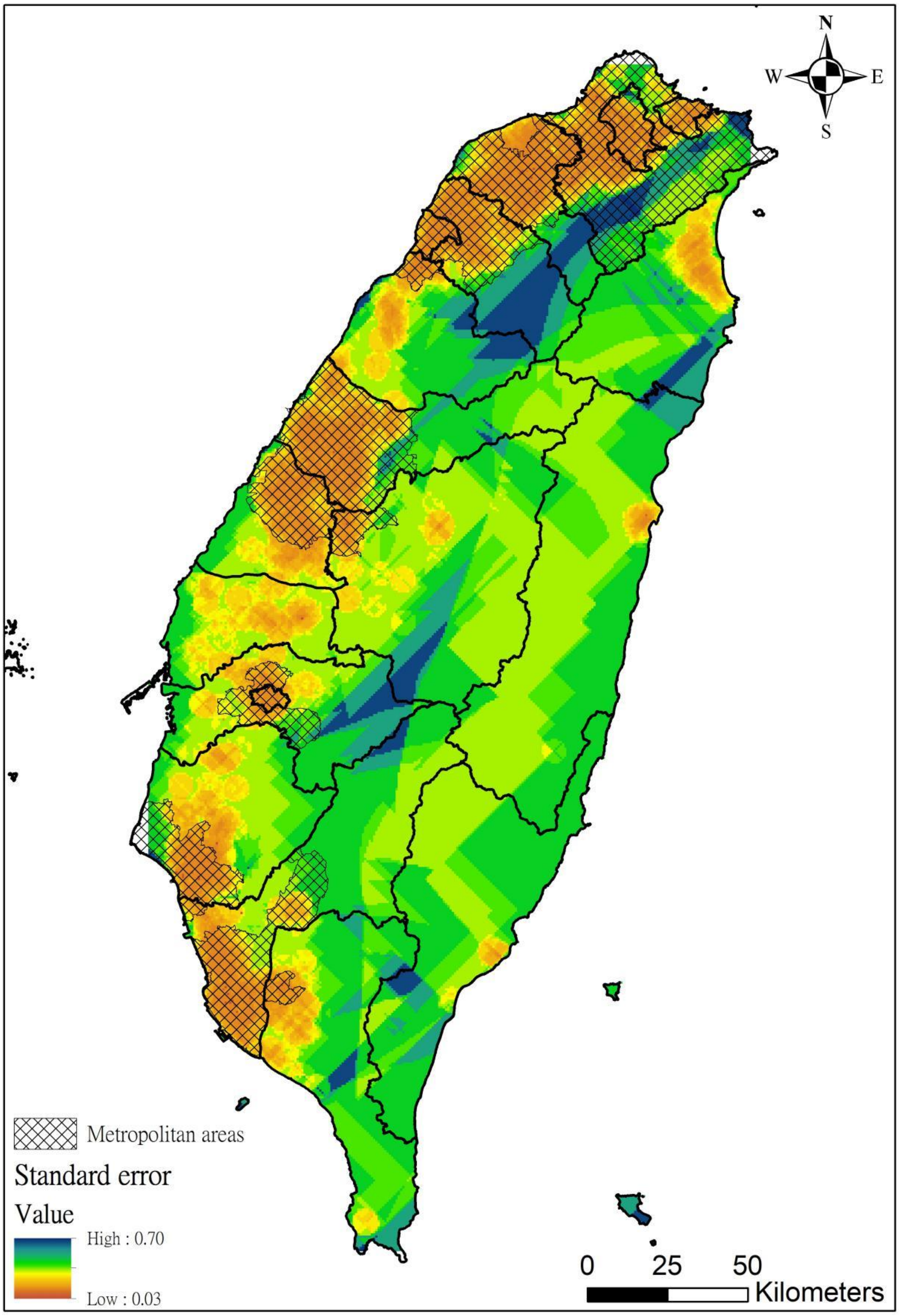

Figure S3. Standard error map of predicted perceived noise.

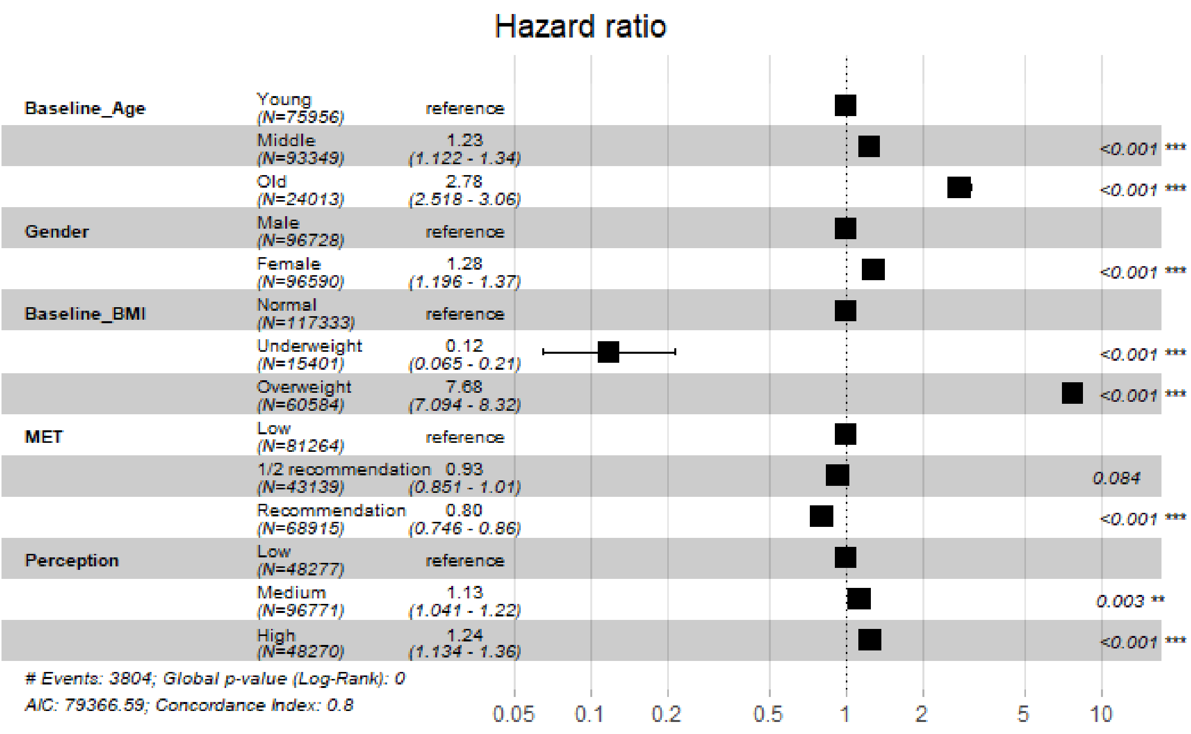

Figure S4. Hazard ratio (and 95% confidence intervals (CI)) of metabolic syndrome (R^2=0.71).

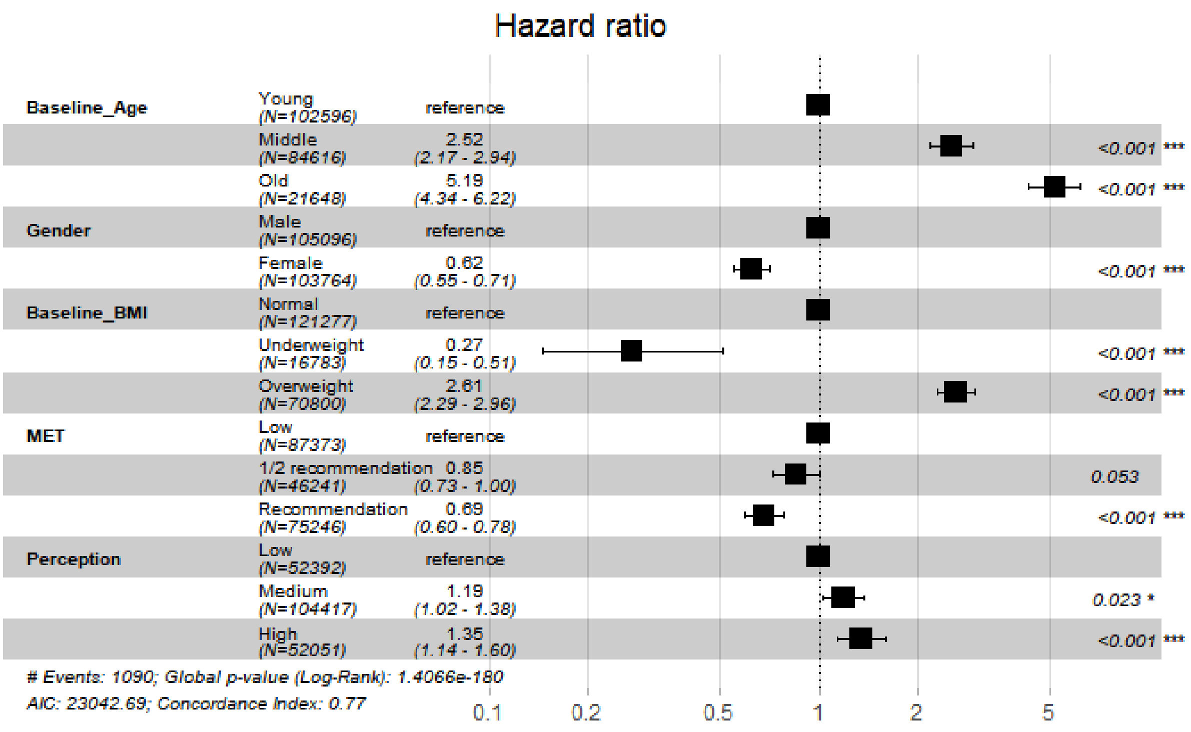

Figure S5. Hazard ratio (and 95% confidence intervals (CI)) of hypertriglyceridemia (R^2= 0.55).

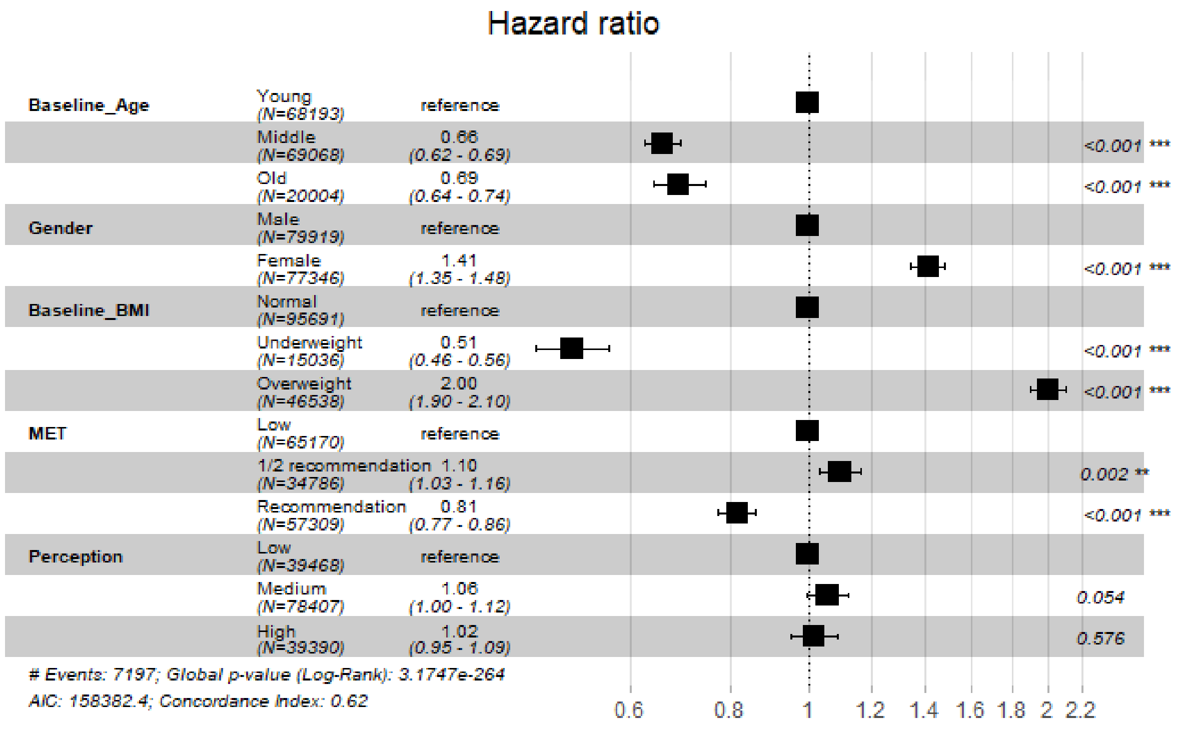

Figure S6. Hazard ratio (and 95% confidence intervals (CI)) of low HDL cholesterol (R^2=0.16).

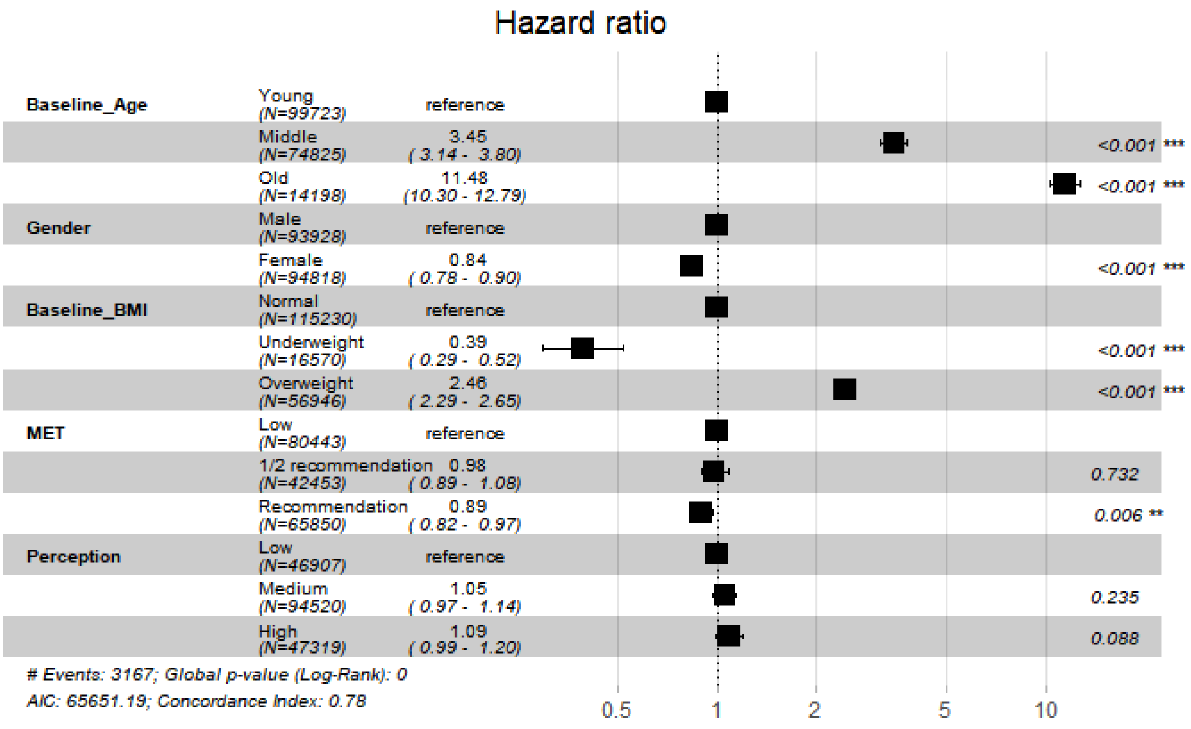

Figure S7. Hazard ratio (and 95% confidence intervals (CI)) of hypertension (R^2=0.50).

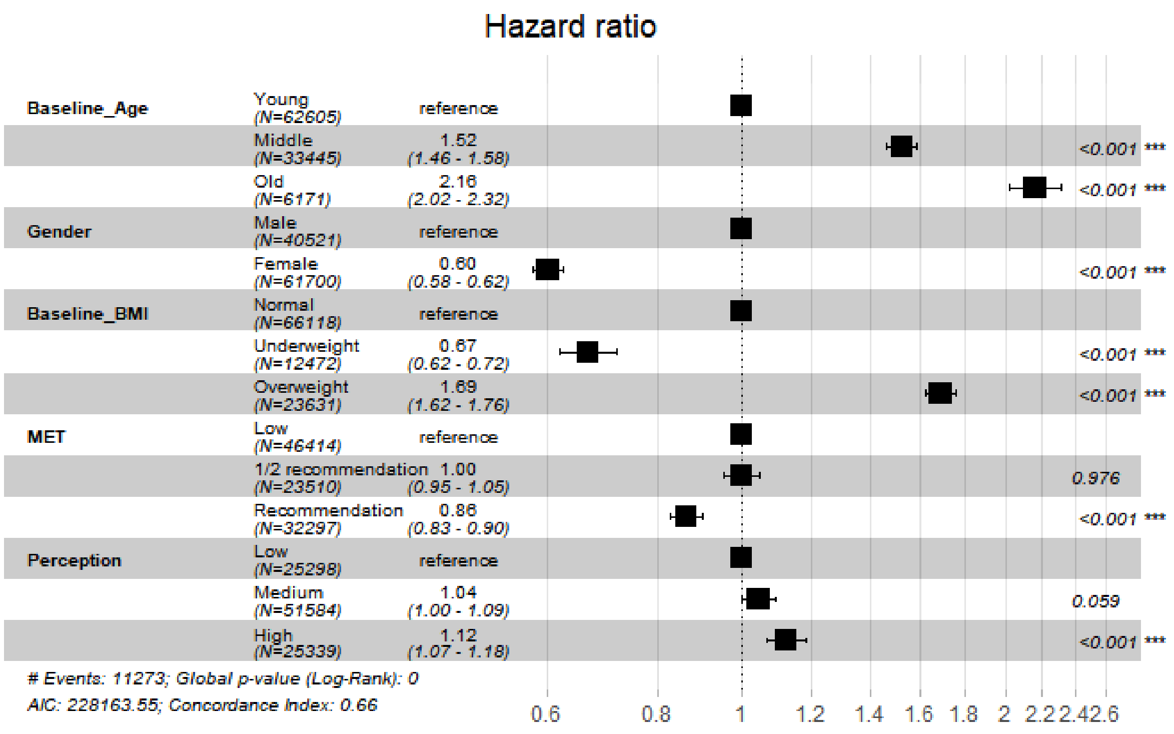

Figure S8. Hazard ratio (and 95% confidence intervals (CI)) of hyperglycemia (R^2=0.23).

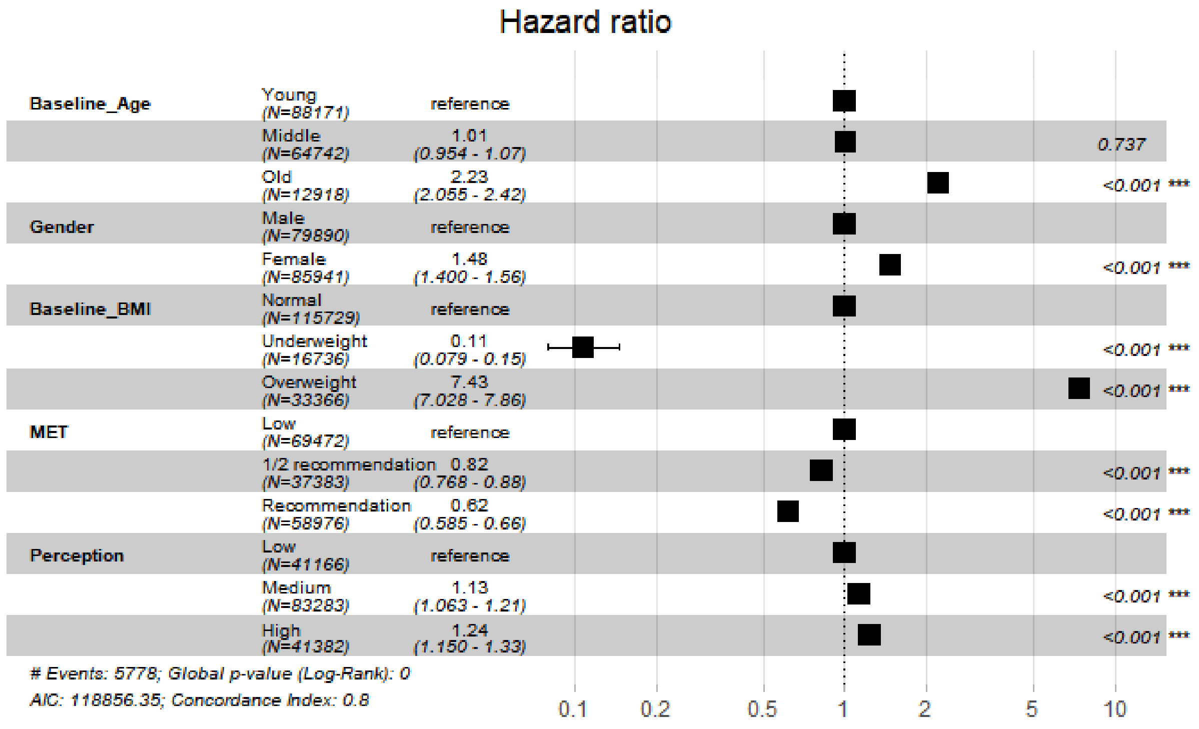

Figure S9. Hazard ratio (and 95% confidence intervals (CI)) of abdominal obesity (R^2=0.68).
